# Challenges in Assessing COVID-19 Vaccines Safety Signals—The Case of ChAdOx1 nCoV-19 Vaccine and Corneal Graft Rejection

**DOI:** 10.3390/vaccines11050954

**Published:** 2023-05-06

**Authors:** Christelle Bizimungu, Martine Sabbe, Françoise Wuillaume, Jamila Hamdani, Philippe Koch, Jean-Michel Dogné

**Affiliations:** 1 DG Post Authorisation—Federal Agency for Medicines and Health Products, 1210 Brussels, Belgium; 2 Department of Ophthalmology, Hôpitaux Iris Sud—Site Ixelles, Réseau Chorus, Université Libre de Bruxelles (ULB), 1050 Brussels, Belgium; 3 Department of Pharmacy, Namur Research Institute for Life Sciences, Namur Thrombosis and Hemostasis Center, University of Namur, 5000 Namur, Belgium

**Keywords:** COVID-19, vaccines, safety, pharmacovigilance, adverse event, causality, corneal graft rejection

## Abstract

The rapid and large-scale roll-out of new COVID-19 vaccines has led to unprecedented challenges in assessing vaccine safety. In 2021, the European Medicines Agency (EMA) processed about 1.7 million safety reports related to COVID-19 vaccines in the EudraVigilance (EV) database and identified more than 900 potential signals. Beyond the large amount of information to be processed, the evaluation of safety signals has faced several difficulties and limitations, both in the assessment of case reports and in the investigation of databases. The evaluation of a signal of corneal graft rejection (CGR) with Vaxzevria^®^ was no exception to this. In this commentary, we present the challenges encountered in making regulatory decisions in the context of evolving evidence and knowledge. The pandemic crisis emphasised the importance of quick and proactive communication to address the many questions and, above all, to ensure the transparency of safety data.

## 1. Introduction

On 21 December 2020, the first COVID-19 vaccine was authorised in the European Union; 12 months later, more than 750 million doses were administered [1]. As with all medicines, vaccines are not 100% safe. Adverse events (AE) that may affect healthy individuals should be promptly identified and assessed. Because of the anticipated rapid and large-scale roll-out of COVID-19 vaccines, the European Medicines Agency (EMA) reinforced the processes for signal detection. Safety signals suggest a new potentially causal association or a new aspect of a known association between a product and an AE. They are generated from various sources such as AEs reported by vaccinees and healthcare professionals, medical events observed in clinical studies and findings published in the scientific literature. In the context of vaccine safety, the purpose of signals assessment is to establish the likelihood of the causal association between an AE and the vaccine of interest. Depending on the results, the assessment may lead the regulatory authorities to take no further action, to request additional analyses or monitoring, to decide on updating the product information and/or the risk management plan of the product or to take urgent safety restrictions including suspension or revocation of a marketing authorisation [2].

In 2021, EMA processed about 1.7 million safety reports related to COVID-19 vaccines in the EudraVigilance (EV) database and identified more than 900 potential signals. Out of these, the Pharmacovigilance Risk Assessment Committee (PRAC) of the EMA validated 21 signals for further investigation [3]. Besides the amount of data to process, the challenges of the assessment of the signals of COVID-19 vaccines are diverse and may include the rarity of a medical event, difficulties in case definitions or the ignorance of the incidence of the event in unvaccinated populations.

In this commentary, we present the challenges encountered as PRAC rapporteur of Vaxzevria^®^ during the assessment of a signal of corneal graft rejection (CGR) with COVID-19 vaccines (Vaxzevria^®^, Comirnaty^®^ and Spikevax^®^) started in April 2022 [4] (Box 1). CGR occurs when the immune system of the graft recipient mistakenly attacks the transplanted cornea. Corneal transplantation is a relatively common procedure and generally has a high success rate. Symptoms of rejection may occur in about 10% of corneal transplants. At the time of signal validation, these COVID-19 vaccines accounted for 24% of all cases of CGR reported to the EudraVigilance database (i.e., 36 cases reported with COVID-19 vaccines out of a total of 148 cases for all medicinal products reported in the EV database). The evaluation of the signal reviewed all available data from the pharmacovigilance databases, the literature and data provided by the marketing authorisation holder.

Box 1 **Challenges and lessons learned in assessing COVID-19 vaccines safety signals. ^1^ AESIs: Adverse Events of Special Interest; ^2^ HCPs: Health Care Professionals.**

Assessing case reports

- Quality and completeness of the information
- Early involvement of identified experts in the investigated fields

Investigating pharmacovigilance databases

- Challenges of disproportionality analyses in the context of massive adverse events reporting and potential masking effect
- Regular update of the list of AESIs ^1^ (including case definitions, e.g., Brighton collaboration)
- Availability of background incidence rates and exposure stratified by region, age group and gender

Deciding despite uncertainties

- Regulatory decision-making in the context of evolving evidence and knowledge
- Experience and awareness of HCPs ^2^ and scientific societies to be taken into consideration
- Delayed data from epidemiological studies

Communication in times of crisis

- Communication also in case of uncertainties and limited evidence
- Being transparent and communicating quickly and proactively

## 2. Assessing Case Reports

The evaluation of case reports consists of reviewing the clinical history and the clinical description of the cases, including the time between the vaccination and the event onset. The medical history allows the identification of alternative or concurrent causes for the event. For example, during the early stages of the vaccination campaign, it was important to be able to identify COVID-19 infections as a potential confounding factor of the event. To standardize the assessment, the likelihood of a causal association is classified using a scale such as the causality assessment system developed by the Uppsala Monitoring Center of the WHO Programme for International Drug Monitoring (WHO-UMC) [5]. This scale evaluates the clinical and pharmacological aspects of a case report as well as the quality of the information. It allows for the classification of the likelihood of the causal relationship but cannot measure it accurately. The benefit of such a scale is contingent on sufficient information on the medical history, the description of the current event and how alternate causes were investigated. Other tools can be used for the review of case reports. For example, the WHO also developed a specific causality assessment algorithm for adverse events following immunization (AEFIs) [6]. Regardless of the scale applied, signal investigators are faced with an initial challenge, the completeness of case reports. 

The evaluation of the signal of CGR comprised the assessment of eighteen cases reports, eleven of which were published in the literature; seven were reported by vaccinees or physicians to the health authorities or pharmaceutical company (the full description of cases is provided in the signal assessment report, available upon request to EMA). All cases occurred in temporal association with the administration of the first or second dose of Vaxzevria^®^, mostly within 3 weeks of the vaccination. Several cases from the literature reported rejection of old grafts which were uneventful for a long period of time and without identifying other local inciting factors. Some of these subjects were at higher risk of rejection, including previous events of CGR, but not all. The evaluation highlighted that the full medical history, including COVID-19 infection, and the full description of the clinical event are often incomplete. This was true for some cases in the literature but even more so for spontaneously reported cases. The limited information made it difficult to ascertain the diagnosis and evaluate potential confounders. The early involvement of an ophthalmologist expert in the evaluation process was important to make the most of the information provided.

After an in-depth review of the cases, the assessors concluded that fifteen reports had a possible causal association, and three reports had a probable causal association according to the WHO-UMC scale.

Overall, the literature cases proved to be more complete and helpful. We should explore how to improve the education of health-care professionals to provide better quality and more complete reports. Avenues for further reflection include the development of key messages describing the information needed, the adaptation of reporting tools with the inclusion of warning messages when information is incomplete, the involvement of scientific societies through contact points, the involvement of specialists acting as spokespeople to stimulate their network, etc.

In times of crisis, it is essential to collect relevant, well-documented, and preferably standardised information as quickly as possible. The follow-up steps are made more difficult by the amount of data to be managed. The reporting tools should preferably be developed in ‘peace time’ for both healthcare professionals and vaccine recipients and their families. They could also benefit outside the context of a large-scale vaccination campaign. 

## 3. Investigating Pharmacovigilance Databases

Two methods use pharmacovigilance databases to identify signals, the disproportionality method and the observed versus expected (O/E) analysis.

The disproportionality method compares the proportion of case reports that include a specific adverse event for the product of interest to the same proportion calculated globally on all case reports for other products. The latter proportion is expected to represent the proportion of the adverse event when no association between the product of interest and the adverse event exists. This method was developed to overcome the impossibility to measure the occurrence of an AE among the users of a product, as the number of users is usually unknown [7].

When applying the method for Vaxzevria and CGR, no statistical disproportionality was found. Yet, experience is lacking in using disproportionality methods when enormous numbers of adverse events are reported, such as in the peak of mass vaccinations against COVID-19. In theory, a sudden overrepresentation of a new product in a pharmacovigilance database can affect the all-product proportions of adverse events in the same database and, as a consequence, the measures of disproportionality for other products. It could lead, for example, to masking a truly disproportionate reporting [8]. In addition, adverse events can be reported differently during a crisis which can also mask a disproportionality signal if there is a lot of reporting noise. Harpaz et al. investigated the masking effect in the context of COVID-19 vaccine signal detection. They found that while masking is rare relative to all possible statistical associations, it is much more likely to occur in COVID-19 vaccine signalling. The statistical signals for AEs related to COVID-19 vaccines, and possibly other vaccines, may go undetected or be delayed due to masking when generated by standard methodologies [9]. These results suggest that masking should be carefully considered when interpreting reports of disproportionality analysis and need further methodological approaches to address this issue.

The core principle of observed versus expected (O/E) analysis is to estimate the expected number of cases occurring by chance alone in vaccinees and compare it with the number of cases reported under the null hypothesis of no association with the vaccine. These analyses are useful tools to confirm a safety signal [10]. They are, however, not sufficient to confirm a causal association or exclude a risk. In the CGR signal evaluation, an O/E analysis did not identify any imbalance. However, the analysis was limited by several uncertainties and assumptions [11].

The expected number of cases is estimated using the background CGR incidence rate, which is determined by the population corneal transplant rate and the rejection rate of grafted eyes. Geographical and temporal variations [12,13], as well as differences related to the type of keratoplasty, should be considered [14]. For example, the transplant rate was estimated to be 5 per 100,000 in the UK versus 2.2 per 100,000 in India, the two countries that account for most of the cases included in the O/E analysis [12]. A rising trend in annual number of transplantations was also observed over the last 30 years [13].

The expected number of cases also depends on the size of the population vaccinated with the vaccine under study. Ideally, vaccination coverage data stratified by age, gender and region should be used; however, these data are not readily available for all countries.

At the same time, the number of observed cases is based on the number of cases reported to the database and not the number of AEs actually occurring in the vaccinated population. The level of underreporting for a given event is unknown and may depend on the awareness and motivation of potential notifiers. A different sensitivity analysis using several levels of underreporting may be suggested for O/E analysis. 

Another parameter of interest for estimating the observed number of cases is the duration of the period at potentially higher risk following immunization. Although a few cases of CGR have been previously reported after vaccination with non-COVID vaccines, such events were poorly characterized in the context of vaccinations [15].

While for CGR, the O/E analysis was of limited value, this type of analysis has proven to be key for the detection of other signals, especially for adverse events of special interest (AESIs). In 2020, before the introduction of COVID-19 vaccines, EMA decided on a list of AESI for the pharmacovigilance of COVID-19 vaccines [16]. This list was used to predefine the case definitions, including risk periods, and the background incidence rates for these events in several European databases. This preparatory work was very beneficial for the monitoring of AESIs in the early phases of the vaccination campaigns. Regular updates of the AESI list, including used case definitions (e.g., Brighton collaboration), remain necessary. Unfortunately, for safety signals dealing with unexpected events, the assessments had to deal with more uncertainties and a lack of accurate background incidence rates adequately stratified by age and geographical regions [17].

## 4. Finding More Evidence

The next step is to identify and understand possible mechanisms of action that can inform the biological plausibility of a causal relationship. In the literature, several hypotheses have been suggested to explain the potential role of COVID-19 vaccines or other vaccines in corneal transplant rejection. However, to date, no definite mechanism has been confirmed [18]. CGR has not been labelled in any product information of vaccines. Furthermore, no epidemiologic studies that investigate a potential association between Vaxzevria^®^ and CGR were published in the literature.

## 5. Deciding despite Uncertainties

Although a causal association has not been confirmed, several authors of published cases proposed risk minimisation measures. These included prophylactic use of corticosteroids in grafted patients and, more particularly, in patients with a high risk of rejection, delay of vaccination or deferring elective corneal transplant to a few months after the complete vaccination course [15].

To recommend any regulatory action, the PRAC has to consider the available evidence and uncertainties around a potential causal association in the context of evolving evidence and knowledge. The experience and awareness of healthcare professionals, as well as the recommendations of medical specialist groups and scientific societies, are also decision parameters. How strong is the evidence? What are the sources of the data (i.e., clinical trials, epidemiological studies, spontaneous/literature case reports, etc.)? How is the medical event managed? What are the recommendations of medical associations for prevention and treatment? These are some of the questions that guide regulatory recommendations.

There is no validated quantitative analysis or algorithm to automatically conclude whether there is a causal association. Conclusions are drawn from case-by-case assessments within the EU system. Signals are discussed at the PRAC level, where representatives of all Member States and experts appointed by the European Commission can comment, and a final conclusion is reached by the PRAC.

In this signal, the PRAC discussion concluded that the overall level of evidence did not support a causal relationship between Vaxzevria^®^ and CGR [19].

Elements supporting a causal association were the few probable cases and the few well-documented episodes of rejection occurring soon after vaccination in grafts, which were uneventful for a long period of time. Other elements did not suggest a causal association and raised uncertainties. Mainly, no imbalance was detected by the disproportionality analysis in EV and by the O/E analysis. The lack of a definite mechanism of action, the absence of a confirmed association between CGR and other vaccines, and the presence of risk factors for rejection in some cases did not help to confirm or rule out a possible causal relationship. 

Moreover, the clinical evaluation, diagnosis and management of CGR are well-established in ophthalmology practice. The signs and symptoms in the cases reported after vaccination with Vaxzevria^®^ were consistent with the classic clinical presentation of CGR, and rejection was appropriately managed with corticosteroids. Furthermore, patients who undergo a corneal transplant are educated to follow up on any symptom indicative of CGR in standard practice. The added value of including a warning and precautions in the product information was considered not warranted.

## 6. Concluding Remarks

In conclusion, the evaluation of the CGR signal did not lead to any regulatory action considering the number of uncertainties that preclude a proper assessment of a causal association. A temporal association is not sufficient to conclude a causal relationship. Sometimes there are no clear answers, and “we don’t know yet” is the only explanation. Progressive insight, clinical studies or epidemiological studies, such as case-control or self-controlled case series (SCCS), may be required to refute or confirm claims of safety concerns attributed to a vaccine. This particularly holds true when the safety issue relates to a disease that occurs with a very low frequency. The Data Analysis and Real World Interrogation Network (DARWIN EU^®^) was recently established by the EMA and the European Medicines Regulatory Network. It supports regulatory decision-making by addressing specific questions through high-quality non-interventional studies independent of industry. DARWIN EU^®^ has already enabled epidemiological studies, such as SCCS, to be carried out in a short period of time.

In addition, continued surveillance activities beyond mass vaccination campaigns remain essential as they can also provide information on the safety of the new vector platform.

And finally, stepwise and proactive communication is required to respond quickly to questions from patients, healthcare professionals and media. Despite the difficulty in explaining the limitations of knowledge, this delicate exercise is essential to ensure the transparency of safety data [20].

## Data Availability

The full signal assessment report on Corneal graft rejection with Vaxervria^®^ (EMA/PRAC/189794/2022) is available on request from EMA website: https://www.ema.europa.eu/en/about-us/contacts/send-question-european-medicines-agency (accessed on 3 May 2023).
